# A MISO-ARX-Based Method for Single-Trial Evoked Potential Extraction

**DOI:** 10.1155/2017/7395385

**Published:** 2017-02-08

**Authors:** Nannan Yu, Lingling Wu, Dexuan Zou, Ying Chen, Hanbing Lu

**Affiliations:** ^1^School of Electrical Engineering and Automation, Jiangsu Normal University, Xuzhou 221116, China; ^2^Department of Internal Neurology, Xuzhou Central Hospital, Xuzhou 221116, China

## Abstract

In this paper, we propose a novel method for solving the single-trial evoked potential (EP) estimation problem. In this method, the single-trial EP is considered as a complex containing many components, which may originate from different functional brain sites; these components can be distinguished according to their respective latencies and amplitudes and are extracted simultaneously by multiple-input single-output autoregressive modeling with exogenous input (MISO-ARX). The extraction process is performed in three stages: first, we use a reference EP as a template and decompose it into a set of components, which serve as subtemplates for the remaining steps. Then, a dictionary is constructed with these subtemplates, and EPs are preliminarily extracted by sparse coding in order to roughly estimate the latency of each component. Finally, the single-trial measurement is parametrically modeled by MISO-ARX while characterizing spontaneous electroencephalographic activity as an autoregression model driven by white noise and with each component of the EP modeled by autoregressive-moving-average filtering of the subtemplates. Once optimized, all components of the EP can be extracted. Compared with ARX, our method has greater tracking capabilities of specific components of the EP complex as each component is modeled individually in MISO-ARX. We provide exhaustive experimental results to show the effectiveness and feasibility of our method.

## 1. Introduction

Evoked potentials (EPs) are localized potential changes generated by the central nervous system when stimulated by well-defined external stimuli (such as electrical, light, sound, and other stimuli) [1]. Thus, EPs can be categorized as auditory evoked potentials (AEPs), visual evoked potentials (VEPs), somatosensory evoked potentials (SEPs), and motor evoked potentials (MEPs) according to the modality of stimulation. Depending on the experimental paradigm, EPs may include a complex of partially overlapping components [2], reflecting different processing stages along the neural pathways. The latency variations of specific components can objectively reflect changes in the underlying state of the neural pathways, which is very meaningful in cognitive science research and clinical applications [3]. Many single-trial EP extracting methods have been proposed in order to enhance the ability to track latency variations.

Parametric modeling using autoregression with exogenous inputs (ARX) is a commonly used method for extracting single-trial EPs over the conventional moving time average [4]. ARX modeling for single-trial EP estimation was first proposed by Cerutti et al. [5]. In ARX, the electroencephalogram (EEG) can be viewed as an autoregression (AR) model driven by white noise, and the EP can be accurately modeled by an autoregressive-moving-average (ARMA) filter with a known signal [6]. The known signal is typically the average of the reference EPs (AREP). The order and parameters of the AR and ARMA models can be estimated by utilizing various optimization techniques, such as the final prediction error (FPE) [5] and the least-squares (LS) method [7]. The EPs can then be reconstructed by ARMA filtering with the AREP. ARX modeling has been widely adopted by researchers to rapidly extract middle latency AEPs, VEPs, and SEPs. For example, Mainardi et al. [8] used the ARX model to quantify changes in auditory N100 for the monitoring of sedation in cardiac surgery patients. Rossi et al. [9] extracted single-trial SEPs with ARX filtering for monitoring the functional integrity of the spinal cord during surgery. Lange and Inbar [10] further extended the ARX estimator to make the single-trial estimation process resistant to noise present in the system using a robust evoked potential estimator (REPE). However, Cerutti et al. [5] recently found, by systemic experimentation, that EP extraction using ARX modeling is completely invalid when latency varies greatly compared with the AREP. We carried out a further study on the single-trial extracting experiments made by De Silva et al. [11]. We found that they always assumed temporal lag between the input and the output of the ARX model equaled zero, which causes significant error when latency varies greatly. In addition, they limited the ARX method to yield a waveform similar to the average response, differing only in global latency. Thus, their procedure cannot demonstrate the method's tracking capabilities of specific components of the EPs. An EP complex may contain components that originated from different functional brain sites [12]. The summation of these components results in component overlap, which may cause partial occlusion of the desired component's features. Because of this, the tracking of latency variations of specific components is very difficult.

In this paper, we present a novel single-trial evoked potential estimation method based on multiple-input single-output ARX (MISO-ARX). In MISO-ARX, each component of the EP is individually modeled by an ARMA filter with a reference signal to avoid different components interfering with each other as in ARX. In addition, all parameters are calculated synchronously to guarantee that the estimated EP is optimal overall. Moreover, as EPs have been proven (in our previous paper) to have strong sparsity over an appropriate dictionary, we first roughly estimate the temporal lag of specific components with sparse coding before calculating the parameters of MISO-ARX in order to improve robustness against great latency variations. A series of experiments carried out on simulated and human test responses confirmed the superior performance of our MISO-ARX method for tracking latency variations even in situations of extremely low SNR. The rest of this paper is organized as follows.  Section 2 gives a detailed description of our single-trial estimation algorithm.  Section 3 contains our experimental results obtained by using the MISO-ARX method and a comparison with ARX and REPE methods.  Section 4 presents our conclusions.

## 2. Single-Trial Evoked Potential Extraction with MISO-ARX

EPs are always embedded in the ongoing spontaneous EEG background, and the SNR is extremely low (below 0 dB). The main parts of our method consist of removing the EEG *e*(*t*) from the measurement *y*(*t*) and then reconstructing the single-trial EP *s*(*t*) [13]. The measurement *y*(*t*) is

(1) $$\begin{array}{c} y\left(\;t\;\right)=s\left(\;t\;\right)+e\left(\;t\;\right). \end{array}$$

### 2.1. The EP Signal

The single-trial EP is considered as a complex containing many components; these components may originate from different functional brain sites and can be distinguished according to their respective latencies and amplitudes. The EP waveform *s*(*t*) is assumed to be a superposition of *Q* components:

(2) $$\begin{array}{c} s\left(\;t\;\right)=\overset{Q}{\underset{q=1}{\sum }}k_{i}v_{q}\left(\;t-\tau_{q}\;\right), \end{array}$$

where *v*_*q*_(*t*) is the basic shape of the *q*th component, *τ*_*q*_ is the component's latency, and *k*_*q*_ indicates the component's amplitude.

In MISO-ARX, each component *v*_*q*_(*t*) is derived by filtering the reference *u*_*q*_(*t*) using the ARMA model parameters, as shown in

(3) $$\begin{array}{c} v_{q}\left(\;t\;\right)=\frac{B_{q}\left(z^{-1}\right)}{A_{q}\left(z^{-1}\right)}u_{q}\left(\;t\;\right), \end{array}$$

where *A*_*q*_(*z*^−1^) = 1 − ∑_*i*=1_^*n*^*q*^^*a*_*i*_^*q*^*z*^−*i*^ and *B*_*q*_(*z*^−1^) = *z*^−*d*^*q*^^∑_*j*=0_^*m*^*q*^−1^*b*_*j*_^*q*^*z*^−*j*^. Thus, (2) can be rewritten as

(4) $$\begin{array}{c} s\left(\;t\;\right)=\overset{Q}{\underset{q=1}{\sum }}k_{q}v_{q}\left(\;t-\tau_{q}\;\right)=\overset{Q}{\underset{q=1}{\sum }}k_{q}\frac{B_{q}\left(z^{-1}\right)u_{q}\left(t-\tau_{q}\right)}{A_{q}\left(z^{-1}\right)}. \end{array}$$

In (4), *k*_*q*_*B*_*q*_(*z*^−1^)*u*_*q*_(*t* − *τ*_*q*_) can be parameterized as

(5) kqBqz−1uqt−τq=kq∑j=0mq−1bjquqt−j−dq−τq=∑j=0mq−1bjqkquqt−j−dq+τq.

Then, we assume ${\overset{-}{b}}_{j}^{q}=b_{j}^{q}k_{q}$ and ${\overset{-}{d}}^{q}=d^{q}+\tau_{q}$, which yields

(6) kqBqz−1uqt−τq=∑j=0mq−1bjqkquqt−j−dq+τq=∑j=0mq−1b−jquqt−j−d−q.

Thus, $k_{q}B_{q}\left(z^{-1}\right)u_{q}(t-\tau_{q})={\overset{-}{B}}_{q}\left(z^{-1}\right)u_{q}(t)$, and we obtain

(7) $$\begin{array}{c} s\left(\;t\;\right)=\overset{Q}{\underset{q=1}{\sum }}k_{q}v_{q}\left(\;t-\tau_{q}\;\right)=\overset{Q}{\underset{q=1}{\sum }}\frac{{\overset{-}{B}}_{q}\left(z^{-1}\right)u_{q}\left(t\right)}{A_{q}\left(z^{-1}\right)}. \end{array}$$

### 2.2. Reference Signal for Each Component

**Figure 1:**
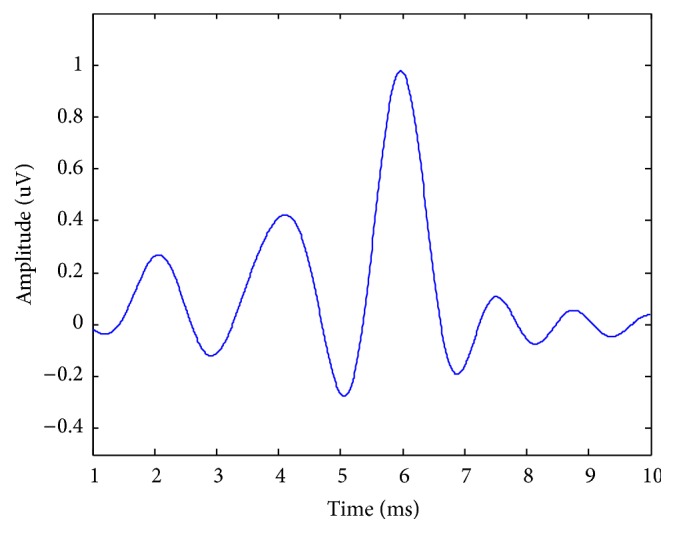
The reference signal extracted from the AREP.

The selection of reference signals, which directly affects the accuracy of EP extraction, is a very important process. In MISO-ARX, we extract the reference signal *u*_*q*_(*t*) for each component from the AREP using a specific filtering window function, such as the Hamming window or the Blackman window. The central location and width of the window are determined by the location and width of the peak, respectively. An example of a simulated decomposition is provided in Figure 1. It can be seen that the simulated waveform consists of three underlying components, which were extracted using a Hamming window.

### 2.3. The EEG Signal

In this paper, the EEG signal *e*(*t*) is viewed as an AR model driven by white noise *w*(*t*), defined as

(8) $$\begin{array}{c} e\left(\;t\;\right)=\frac{1}{A_{e}\left(z^{-1}\right)}w\left(\;t\;\right), \end{array}$$

where *A*_*e*_(*z*^−1^) = 1 − ∑_*i*=1_^*n*^*e*^^*a*_*i*_^*e*^*z*^−*i*^.

### 2.4. Estimation of Temporal Lag

In ARX, most researchers assume that temporal lag *d* between the input and output of the model is equal to zero before estimating the model orders *m* and *n*. This assumption is not in accordance with practice. In this study, we used sparse coding to roughly estimate the value of *d*. Sparse coding has had significant success in signal denoising and separation. In addition, in our previous paper, EPs were proven to have strong sparsity over an appropriate dictionary [14]. Assuming *D* and *θ* are the dictionary and the sparse coefficients, respectively, *s*(*t*) can be expressed as *s*(*t*) = *Dθ* [15]. Thus, *y*(*t*) is

(9) $$\begin{array}{c} y\left(\;t\;\right)=s\left(\;t\;\right)+e\left(\;t\;\right)=D\theta +e\left(\;t\;\right). \end{array}$$

The estimator for *θ* is calculated by solving

(10) θ^=arg minθ θ0s.t. yt−D·θ2≤ε0,

where *ε*_0_ is determined by the variance of the EEG. Equation (10) can be solved by using optimization methods, such as basis pursuit [16], orthonormal matching pursuit [17], and Lasso [18]. Since the atoms of *D* are constructed by left or right translation of the basic components of EPs, we use the location of nonzero values in *θ* to estimate the temporal lag *d*.

### 2.5. Single-Trial Extraction

By replacing (7) and (8) in (1), we get

(11) ytst+et=∑q=1QB−qz−1uqtAqz−1+1Aez−1wt.

In order to simplify this model, we assume *A*_*q*_(*z*^−1^) = *A*_*e*_(*z*^−1^) = *A*(*z*^−1^)  . This implies a partial loss of generality with respect to the AR model for the noise and the ARMA model for the signal, completely independent of each other. Nevertheless, the MISO-ARX model requires more complex algorithms to be characterized. In this paper, this MISO system is characterized with the global separable nonlinear multi-innovation recursive least-squares-identification method [19]. Then, parameters *A*(*z*^−1^) and ${\overset{-}{B}}_{q}(z^{-1})$ are estimated. The EP can be reconstructed as

(12) $$\begin{array}{c} s\left(\;t\;\right)=\overset{Q}{\underset{q=1}{\sum }}s_{q}\left(\;t\;\right)=\overset{Q}{\underset{q=1}{\sum }}\frac{{\overset{-}{B}}_{q}\left(z^{-1}\right)u_{q}\left(t\right)}{A\left(z^{-1}\right)}. \end{array}$$

## 3. Experimental Results

### 3.1. Simulation Experiment

A computer simulation was conducted to verify the performance of our MISO-ARX method for EP signal estimation. Three single-trial-estimation methods for EP signals, namely, ARX, REPE, and SP, were compared in the following simulations. In order to measure the performance of each of the methods, we measured the SNR of the estimated EPs and the accuracy of the latencies for different SNRs to evaluate the quality of these methods. All experiments were implemented in Matlab R2006b on a Pentium 2.7 GHz PC with 4 GB RAM.

The reference signals *v*_*i*_(*k*) were simulated by the superimposition of three basic components, which can be represent by the Gaussian distribution function [20]; thus

(13) ut=0.25sinc⁡0.13π4t−8+0.5sinc⁡0.13π4t−16+sinc⁡0.13π4t−24+l.

**Figure 2:**
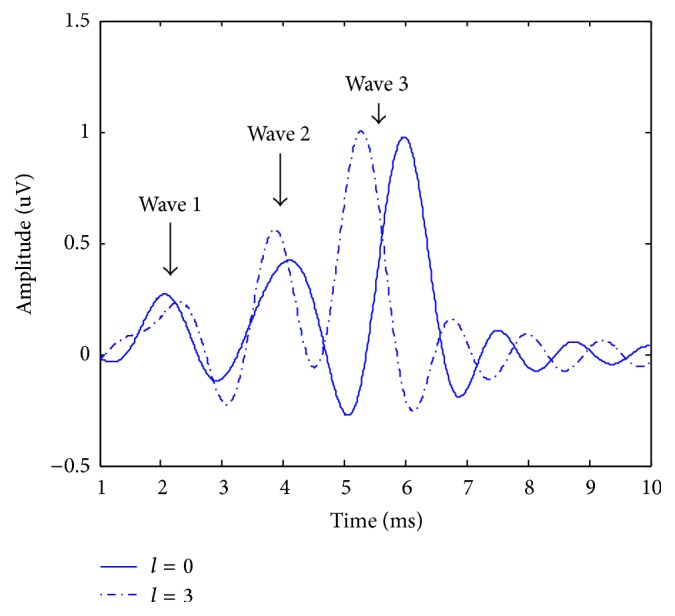
The waves of the reference signals in *l* = 0 and *l* = 3.

The synthetic reference signals (*l* = 0 and *l* = 3) are shown in Figure 2.

**Figure 3:**
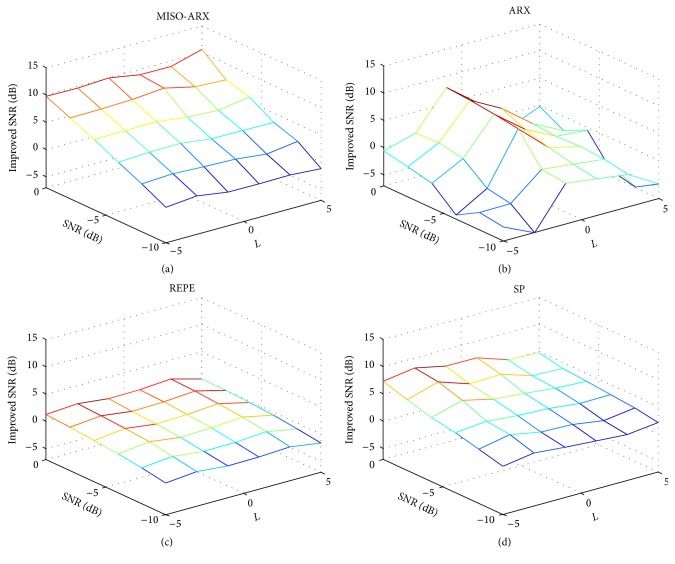
Performance evaluation of four methods with different SNR and *l* values.

From Figure 2, it can be seen that all three components have changed and the latency of the third wave varies significantly. In this study, we used an EP with *l* = 0 as the reference signal. The background EEG superimposed on the EP signal was simulated by an autoregressive process [21], as shown in the following equation:

(14) qt=1.5084qt−1−0.1587qt−2−0.3109qt−3−0.0510qt−4+wt,

where *w*(*t*) is Gaussian white noise. During the process of estimation, the SNR of the observations may change over time due to the nonstationary characteristics of the EEG. Therefore, in this experiment, the performance of the four different methods was examined under various SNR conditions. The SNRs of the observations were changed from 0 dB to −10 dB, and *l* was changed from −5 to 5. For each SNR value, 100 pairs of observations were generated. The average results for different SNR and *l* values in 100 independent runs are shown in Figure 3.

**Figure 4:**
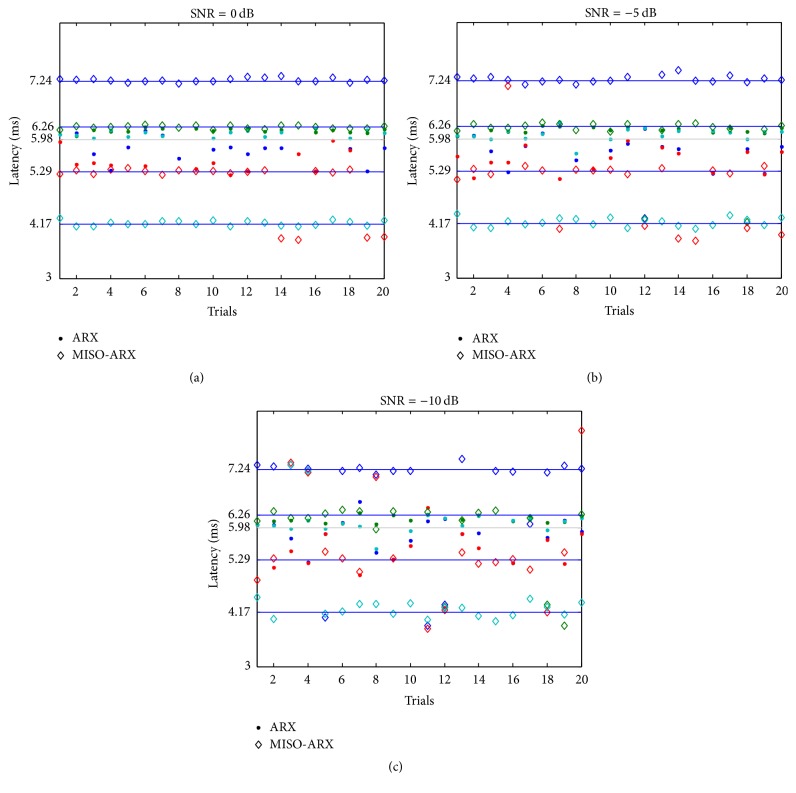
Latency tracking of the third wave using ARX and MISO-ARX.

It is clear that, with the decrease of the value of the SNR, estimation performance declines. However, in MISO-ARX, the changes in *l* had hardly any impact on the estimation results. This illustrates that our method is apt for tracking latency variation of EPs. Since the SNR is defined by the complete signal and not by a specific feature of it, we measured the MISO-ARX method's ability of tracking latency variations. We compared ARX and MISO-ARX in the case of three different SNR values (0 dB, −5 dB, and −10 dB) and four different latencies (4.17 ms (*l* = 7), 5.29 ms (*l* = 3), 6.26 ms (*l* = −1), and 7.24 ms (*l* = −5)). Results are shown in Figure 4. We can see from Figures 4(a) and 4(b) that, for high SNR values (0 dB and −5 dB), our method had strong tracking capability for all latencies.  Figure 4(c) indicates a deterioration of latency tracking performance when the SNR decreased, but the MISO-ARX method's accuracy rate still exceeded 70%. With ARX, even for high SNR values, for *l* = −5 and *l* = 7 the estimations were totally wrong, suggesting that tracking such variations is not possible.

### 3.2. Real Data

For further evaluation of the performance of our method, VEPs were collected from six eyes belonging to three human subjects during pattern reversal VEP experiments. This study was conducted with the approval of the local ethics committee, and all experiments with human participants were performed according to the National Institutes of Health Guidelines. VEP signals were recorded via a surface electrode placed on the occipital region of the scalp (Oz), using the right earlobe as a reference, with the forehead grounded. Subjects were required to gaze at a cross on the stimulus screen. The stimulus pattern was a conventional black-and-white checkerboard, which was reversed every half a second. Recordings were made using a digital EEG recording system (NuAmps EEG Amplifier, NeuroScan, USA) with a sampling rate of 1000 Hz and stored on a computer. Signals were bandpass filtered in the range of 0.05–450 Hz.

**Figure 5:**
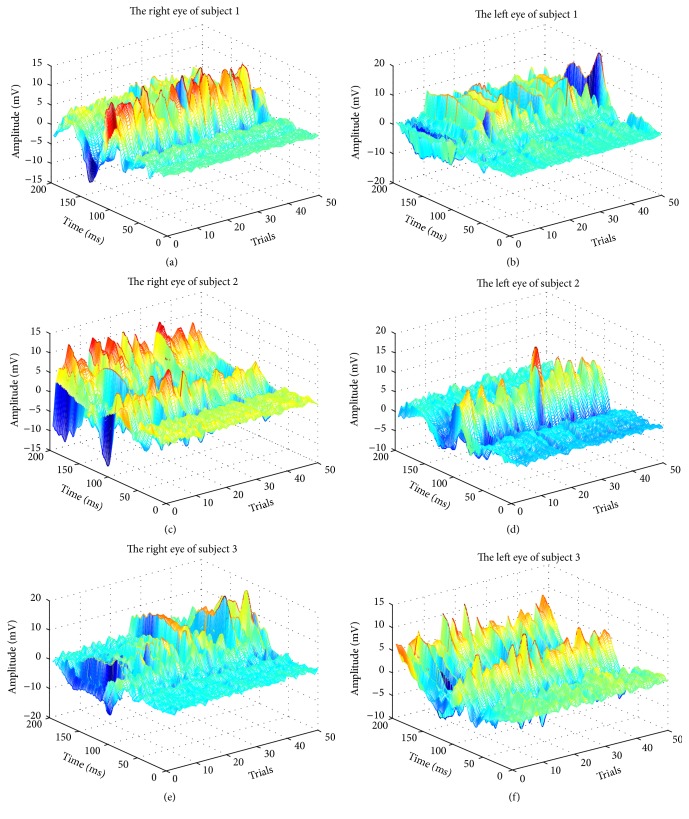
Estimation performance.

In the VEP signal, critical responses were located at approximately 100 ms after stimulation, where the positive peak (P100) occurred. With the stimulus being delivered at 0 ms, we processed each VEP trial from 0 ms to 200 ms. We used data from the first 50 pairs of trials to perform the experiment, from six eyes belonging to three subjects, as shown in Figure 5. As shown in the figure, it is clear that most estimated VEPs have a peak at around 100 ms.

**Figure 6:**
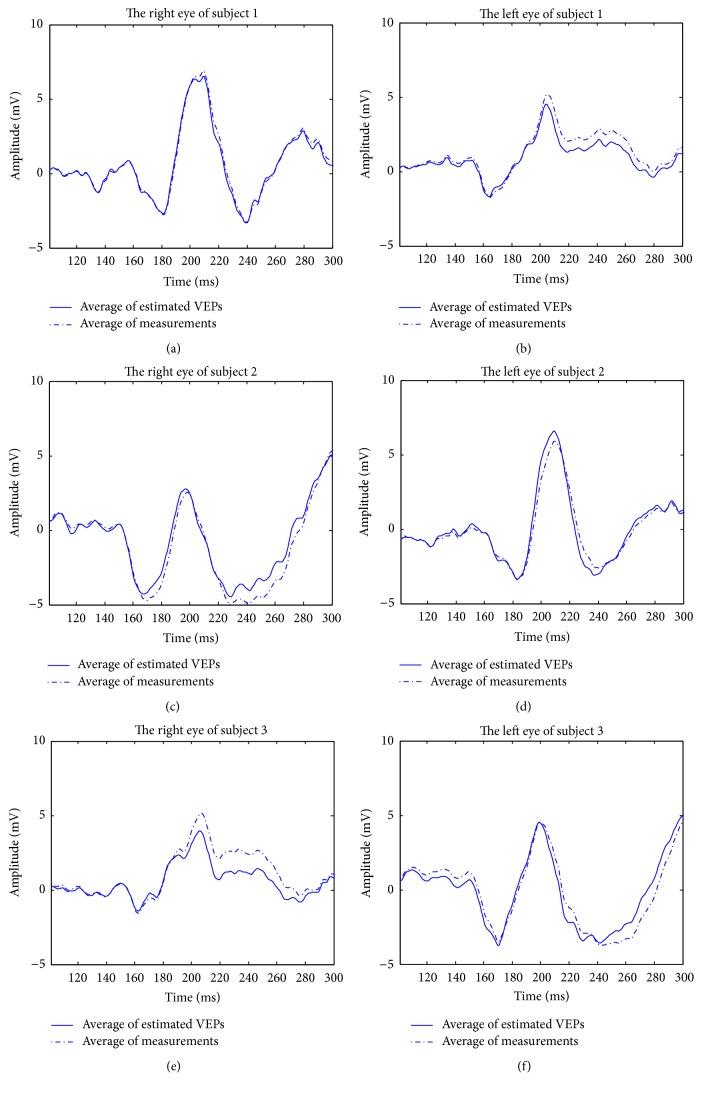
Average of the estimated VEPs and the average of the measurements.

For each eye, we used data from the first 50 pairs of trials to calculate the average of the estimated VEPs. The results are shown in Figure 6. The estimates using MISO-ARX are indicated by solid line, and the averages of the measurements are indicated by dashed-dotted line. Clearly, as shown in Figure 6, the average of the estimated results for each eye is very similar to the average of the measurements.

**Figure 7:**
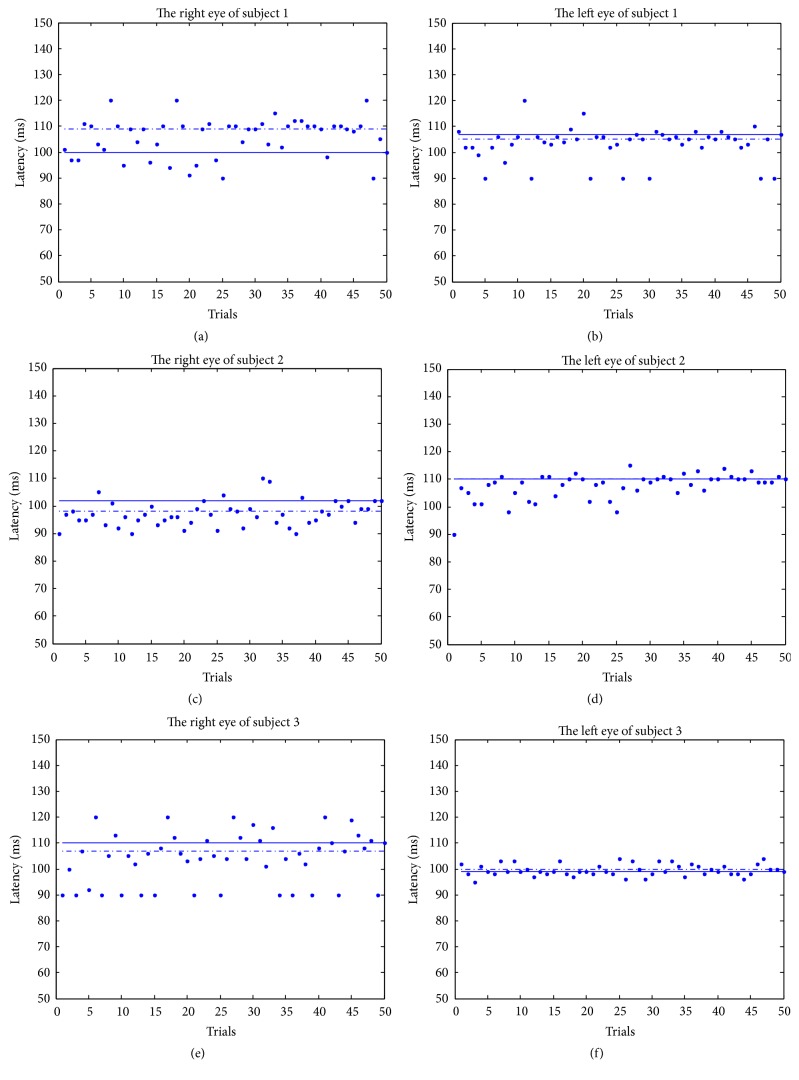
Estimation of latencies of P100 by MISO-ARX.

Then, we estimated the latency of the P100 for each trial, as shown in Figure 7. The estimates are indicated by dots, the averages of the estimates are indicated by solid line, and the P100 latencies of the average of the measurements are indicated by dashed-dotted line. We can see that the trial-to-trial variation in the latencies of P100 is large. However, the averages of the estimates are close to the results of the average of the measurements.

## 4. Conclusion

In this paper, we presented a novel single-trial EP extraction method based on MISO-ARX. This method considers the single-trial EP as a complex containing many components, and each component can be modeled by ARMA. In addition, in order to improve the accuracy of the model parameters estimation, we used sparse coding to roughly estimate temporal lag. Since each component is modeled individually, our method has greater tracking capabilities of specific components of the EP complex. We conducted a series of experiments on synthetic and real data, and the results were evaluated using waveform observations and several metrics. From point of view of the experimental results, our method achieved a better and more favorable estimation performance than other currently used state-of-the-art methods in single-trial EP estimations.

## References

[1] Wang Q., Wu Y., Liu W., Gao L. (2016). Dominant eye and visual evoked potential of patients with myopic anisometropia. *BioMed Research International*.

[2] Gevins A. S. (1984). Analysis of the electromagnetic signals of the human brain: milestones, obstacles, and goals. *IEEE Transactions on Biomedical Engineering*.

[3] Costa M. H. (2012). Estimation of the noise autocorrelation function in auditory evoked potential applications. *Biomedical Signal Processing & Control*.

[4] Regaçone S. F., Lima D. D. B. D., Valenti V. E., Frizzo A. C. F. (2015). Resting heart rate and auditory evoked potential. *BioMed Research International*.

[5] Cerutti S., Baselli G., Liberati D., Pavesi G. (1987). Single sweep analysis of visual evoked potentials through a model of parametric identification. *Biological Cybernetics*.

[6] Berger B., Minarik T., Liuzzi G., Hummel F. C., Sauseng P. (2014). EEG oscillatory phase-dependent markers of corticospinal excitability in the resting brain. *BioMed Research International*.

[7] Kumru H., Soler D., Vidal J., Tormos J. M., Pascual-Leone A., Valls-Sole J. (2012). Evoked potentials and quantitative thermal testing in spinal cord injury patients with chronic neuropathic pain. *Clinical Neurophysiology*.

[8] Mainardi L. T., Kupila J., Nieminen K. (2000). Single sweep analysis of event related auditory potentials for the monitoring of sedation in cardiac surgery patients. *Computer Methods & Programs in Biomedicine*.

[9] Rossi L., Bianchi A. M., Merzagora A., Gaggiani A., Cerutti S., Bracchi F. (2007). Single trial somatosensory evoked potential extraction with ARX filtering for a combined spinal cord intraoperative neuromonitoring technique. *BioMedical Engineering Online*.

[10] Lange D. H., Inbar G. F. (1996). A robust parametric estimator for single-trial movement related brain potentials. *IEEE Transactions on Biomedical Engineering*.

[11] De Silva A. C., Sinclair N. C., Liley D. T. J. (2012). Limitations in the rapid extraction of evoked potentials using parametric modeling. *IEEE Transactions on Biomedical Engineering*.

[12] Lange D. H., Pratt H., Inbar G. F. (1997). Modeling and estimation of single evoked brain potential components. *IEEE Transactions on Biomedical Engineering*.

[13] Kramer J. L. K., Haefeli J., Curt A., Steeves J. D. (2012). Increased baseline temperature improves the acquisition of contact heat evoked potentials after spinal cord injury. *Clinical Neurophysiology*.

[14] Yu N., Liu H., Wang X., Lu H. (2013). A joint sparse representation-based method for double-trial evoked potentials estimation. *Computers in Biology & Medicine*.

[15] Yu N., Hu F., Zou D., Ding Q., Lu H. (2016). Single-trial sparse representation-based approach for VEP extraction. *BioMed Research International*.

[16] Shental O. Sparse representation of white Gaussian noise with application to L0-norm decoding in noisy compressed sensing.

[17] Chen S. S., Donoho D. L., Saunders M. A. (2001). Atomic decomposition by basis pursuit. *SIAM Review*.

[18] Pati Y. C., Rezaiifar R., Krishnaprasad P. S. Orthogonal matching pursuit: recursive function approximation with applications to wavelet decomposition.

[19] Wang J. H., Wang D. B., Wang Z. S. (2010). Recursive identification of MISO systems with multiple unknown time delays. *Control and Decision*.

[20] Jewett D. L., Williston J. S. (1971). Auditory-evoked far fields averaged from the scalp of humans. *Brain*.

[21] Kamel N., Yusoff M. Z., Hani A. F. M. (2011). Single-trial subspace-based approach for VEP extraction. *IEEE Transactions on Biomedical Engineering*.

